# Odontogenic Cutaneous Fistula in Pregnant Woman: Is Long-Term Antibiotic Therapy the Solution?

**DOI:** 10.7759/cureus.82948

**Published:** 2025-04-24

**Authors:** Hideto Imura, Chi K Nguyen, Teruyuki Niimi, Ryosuke Miwa, Nagato Natsume

**Affiliations:** 1 Cleft Lip and Palate Center, Aichi Gakuin University, Nagoya, JPN; 2 Division of Research and Treatment for Oral and Maxillofacial Congenital Anomalies, School of Dentistry, Aichi Gakuin University, Nagoya, JPN; 3 Special Treatment Department, Odonto-Maxillo-Facial Hospital of Ho Chi Minh City, Ho Chi Minh, VNM

**Keywords:** antibiotic therapy, chronic osteomyelitis, cutaneous facial sinus tract, extra-oral sinus tract, maternal health, ondotogenic cutaneous fistula, post-extraction complication, pregnancy

## Abstract

A 30-year-old pregnant woman developed an odontogenic cutaneous fistula (OCF) five months after having her wisdom tooth extracted. Initially diagnosed as a cutaneous furuncle, the condition was treated with amoxicillin, resulting in temporary improvement. However, it recurred after a month. Upon further evaluation, the lesion was diagnosed as an OCF originating from a chronic dental infection. Due to the patient’s pregnancy, the treatment focused on non-invasive periodontal care, including scaling, while antibiotic therapy was maintained. Amoxicillin was initially prescribed; however, due to the progression of the infection, the treatment was switched to clarithromycin after consultation with a dermatologist. Over five months, the lesion gradually resolved, with no acute symptoms remaining. The pregnancy progressed without complications, and the patient gave birth to a healthy baby. This case underscores the challenges of managing odontogenic infections during pregnancy and highlights the importance of tailored antibiotic therapy to ensure the safety of both the mother and fetus.

## Introduction

Odontogenic cutaneous fistula (OCF) is characterized by a fistulous tract connecting the oral cavity and the skin, caused by a chronic infection originating from a tooth [1]. Nearly 80% of cervicofacial sinuses are of odontogenic origin, with most cases related to periapical-periodontal infection, pericoronitis of the lower wisdom tooth, and chronic osteomyelitis arising from dental infections [2,3]. In most cases, the cutaneous lesions typically appear near the causative teeth [3]. The pathway of infection from dental and surrounding structures to the skin is influenced by the attachment of facial muscles. The infection tends to follow the path of least resistance, which is determined by muscle attachments and the distribution of loose connective tissue in the region [3]. For example, infections originating from the canine teeth seldom lead to sinus formation near the medial angle of the eye, whereas those from maxillary molars, whose roots are located above the buccinator muscle attachment, often result in sinus tracts on the cheek. The clinical characteristics of the lesions have a large variety, including nodules (mostly seen), abscesses, fistulas, gummas, scars, cysts, and ulcers [1-5]. The diversity of lesions and the insidious manifestations of dental infection often make OCF misdiagnosed as a dermatological lesion. Many studies show that nearly 50% of patients with OCF experience delayed diagnosis [1-4]. The unresolved dental cause allows the infection to persist, leading to unnecessary tests and treatments such as biopsies and prolonged antibiotic therapy [2,6]. If left untreated, the fistula often results in persistent, uncomfortable scarring [3,4]. Herein, we present a rare case of OCF in a pregnant woman after a lower wisdom tooth extraction. Pregnancy-related changes may contribute to the pathogenesis and pose challenges in the treatment of this patient.

## Case presentation

A 30-year-old woman underwent left mandibular wisdom tooth extraction without any early complications. Four months later, she experienced a miscarriage. Five months after the extraction, she developed persistent pain and swelling in the left mandibular region with a 5x3 mm elastic, pressure-sensitive nodule. Initially, she was diagnosed with a cutaneous furuncle and received 21 days of amoxicillin treatment at a private dental clinic, which reduced the nodule size to 1x2 mm. However, one month later, the nodule and swelling recurred, prompting a referral for further evaluation. During this time, she became pregnant again.

**Figure 1:**
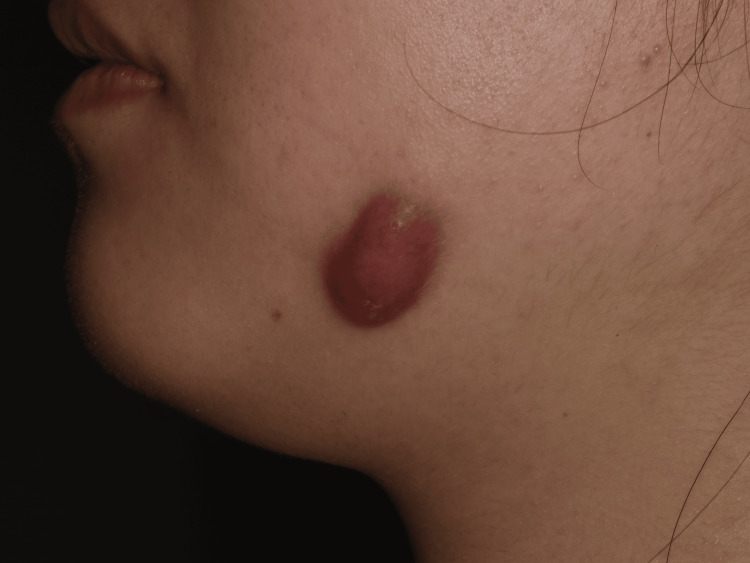
A 10 mm cutaneous lesion in the left submandibular region. The nodule was noted well-defined, with redness, tenderness, and no drainage.

**Figure 2:**
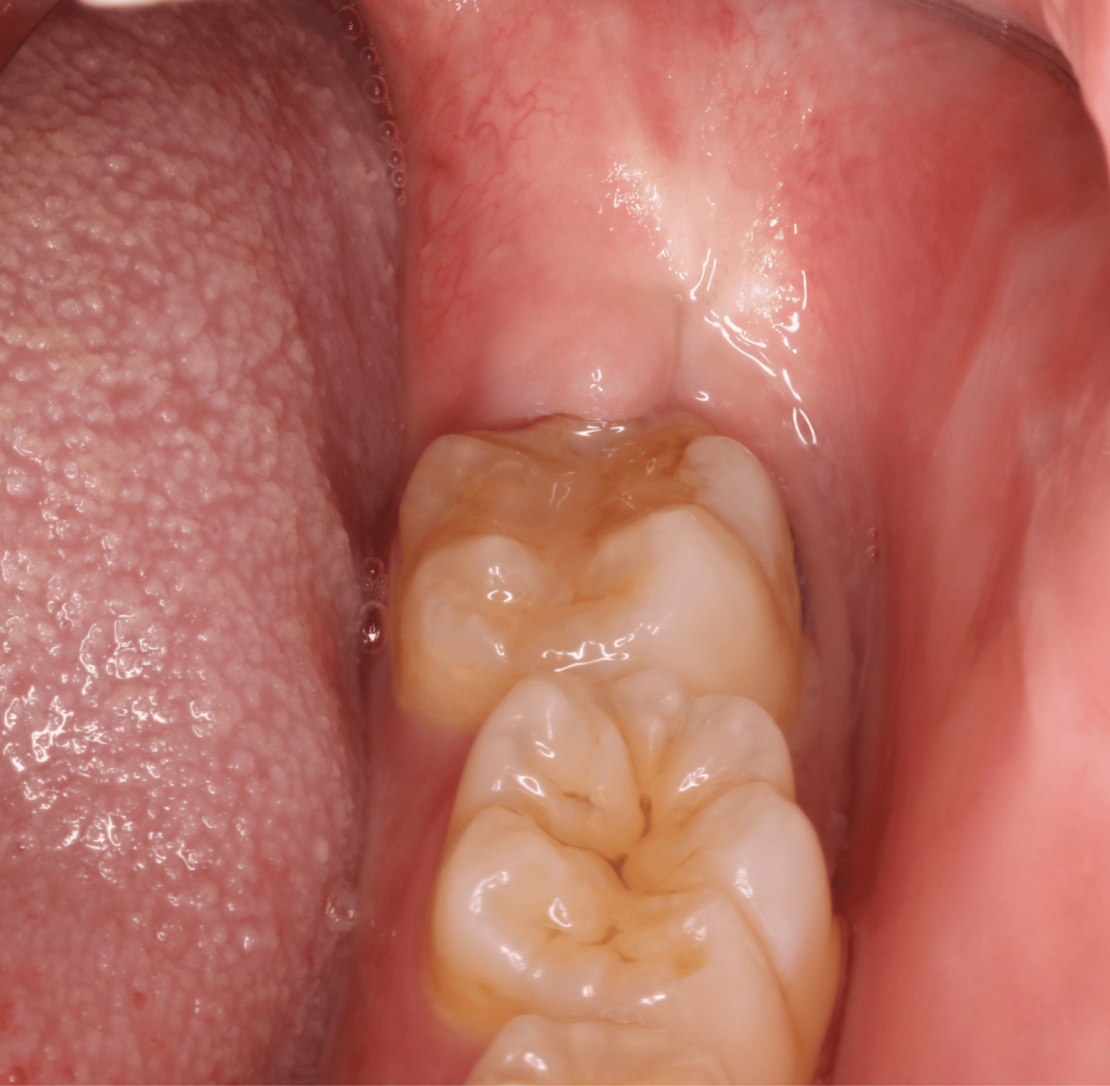
Swollen gum observed at the distal periodontal pocket of the second left molar. The patient reported tenderness but no drainage at the wisdom tooth extraction site.

**Figure 3:**
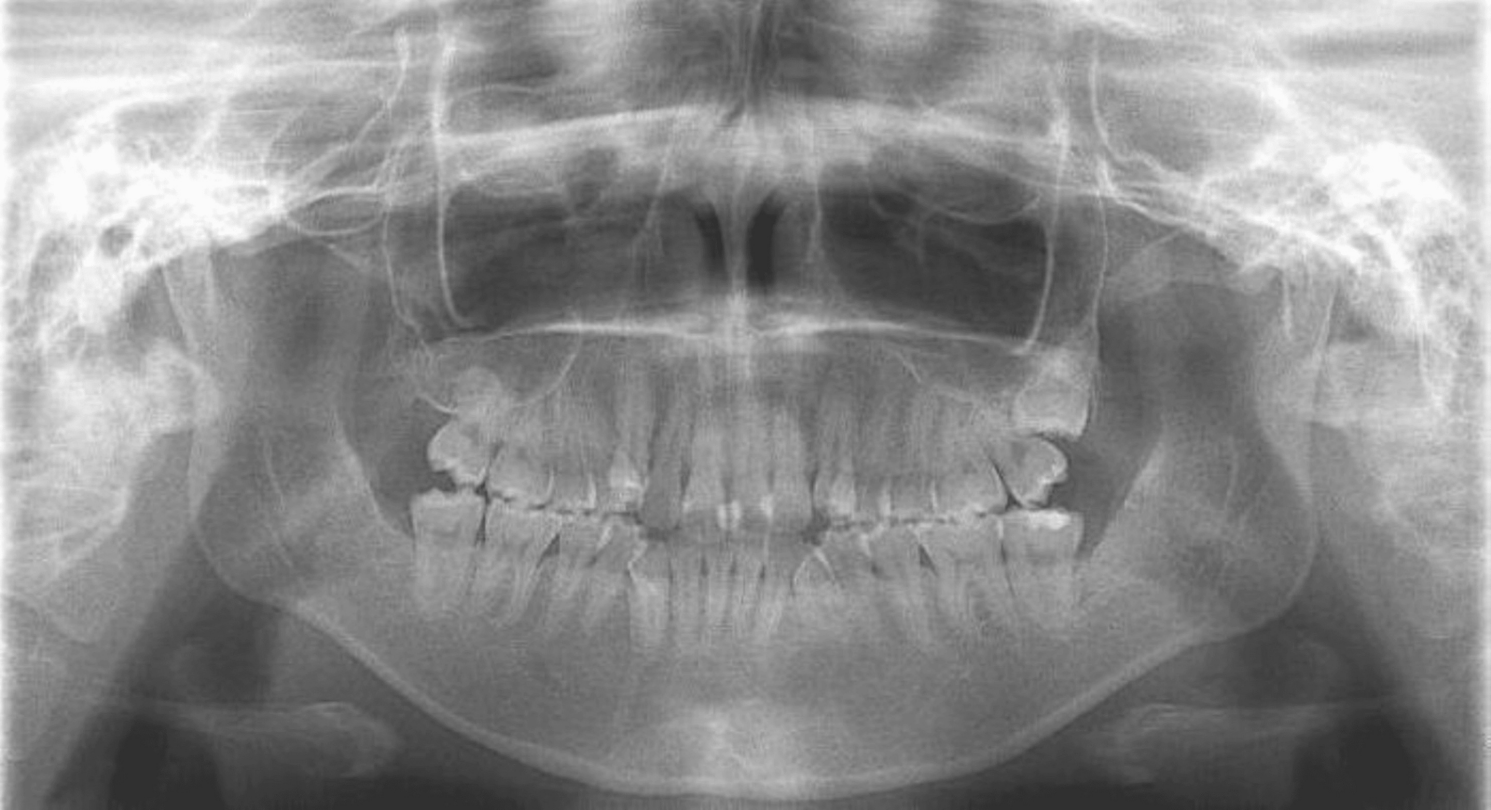
Panoramic X-ray shows no anomalies.

**Figure 4:**
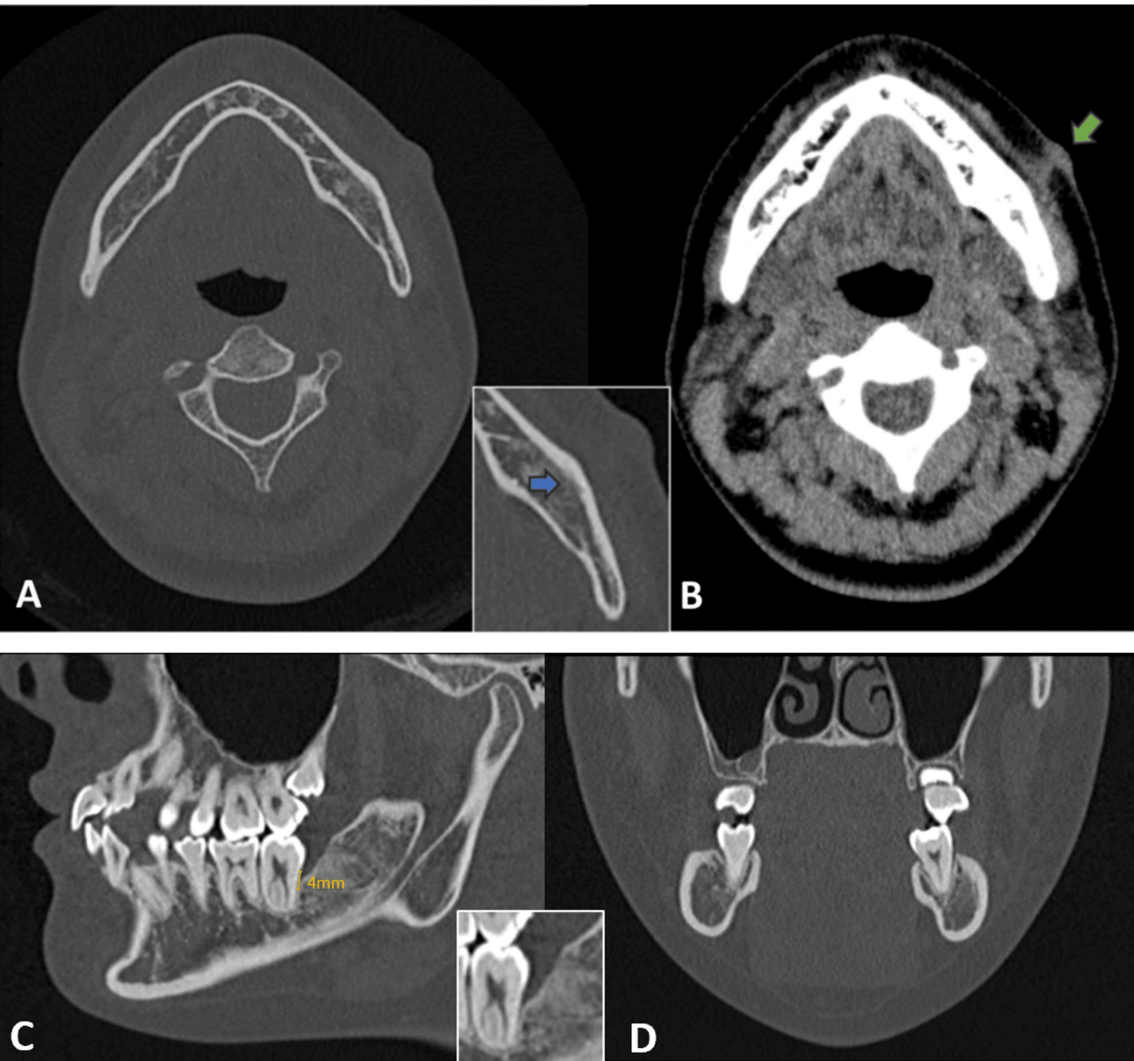
CT imaging. (A) T1-weighted image shows an irregular bone contour (blue arrow), and a slightly thicker outer bone plate may indicate a solid periosteal reaction in the left mandibular region. (B) T2-weighted image shows a fistulous tract connecting the infected site to the cutaneous lesion (green arrow). (C) T1-weighted sagittal plane shows bone loss distal to the second molar, and the distance from the cementoenamel junction to the base of the pocket is 4 mm. (D) T1-weight frontal plane shows that bone loss does not occur at the buccal side.

On her first visit to our hospital, the patient was two months pregnant and presented with a 10 mm external nodule, slightly swollen gums, tenderness at the extraction site, and no discharge (Figures 1,2). The periodontal pocket on the distal side of the second molar is 8 mm. Initially, we suspected osteomyelitis of the left lower jaw and treated her with amoxicillin for 7 days. Two weeks later, an 11 mm lesion (larger, dark purplish, swollen, with pain on pressure) was noted, indicating infection progression. Blood tests showed mild inflammation (CRP: 2.4 mg/dL, reference range: 0-1 mg/dL; neutrophils: 74.4%, reference range: 37.0-72.0%; lymphocytes: 18.4%, reference range: 15.0-50.0%). There were no abnormalities on the panoramic film (Figure 3), but CT imaging revealed a fistulous connection between the cutaneous lesion and the extraction site, confirming OCFs (Figure 4). Since the lesion did not have any exudate or pus, we were unable to perform pus culture and antibiotic sensitivity testing on this patient. Additionally, the patient did not agree to undergo aspiration of the facial lesion. Treatment decisions were primarily based on the progression of the clinical symptoms. Due to her pregnancy, we limited surgical intervention, focusing on periodontal scaling of the surrounding area while continuing amoxicillin treatment for five days.

**Figure 5:**
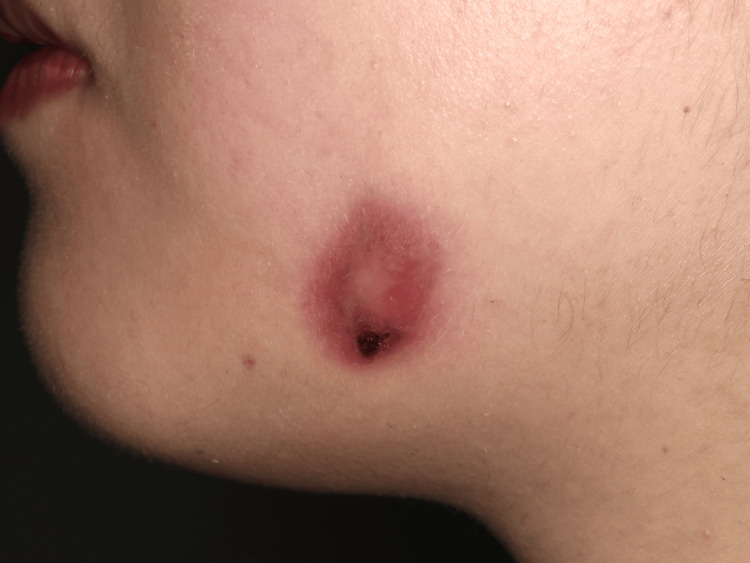
By three months post-consultation, the swelling and redness had reduced, and the lesion developed central drainage, suggesting healing.

Three months after the first visit, the swelling and redness had reduced, and the lesion developed central drainage, suggesting healing (Figure 5). She entered her second trimester, and after consulting with a dermatologist, her treatment switched to Clarithromycin 200 mg (two capsules daily for 14 days). At four months post-consultation, pain and swelling significantly reduced, and she was continuously prescribed Clarithromycin for 30 days. Curettage was planned postpartum if necessary. Five months after the first visit, the lesion had completely resolved, antibiotics were discontinued, and no acute symptoms remained.

**Figure 6:**
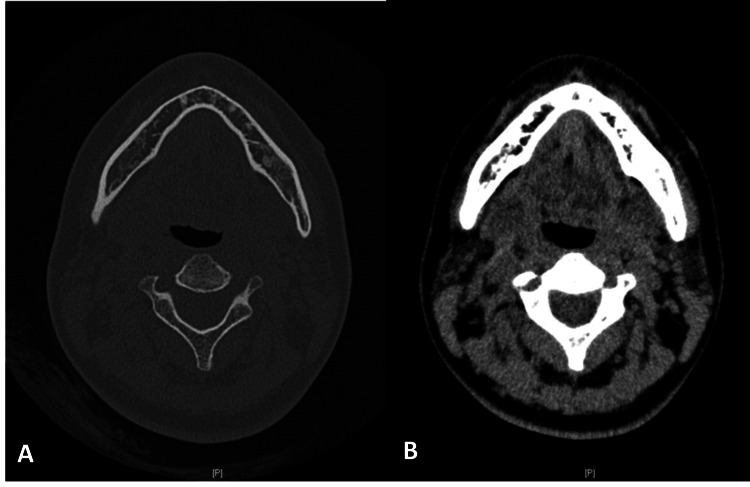
Post-treatment CT reveals significant healing. The fistulous connection in the left mandibular region has faded, and the nodule gradually disappeared. (A) T1-weighted image. (B) T2-weighted image.

**Figure 7:**
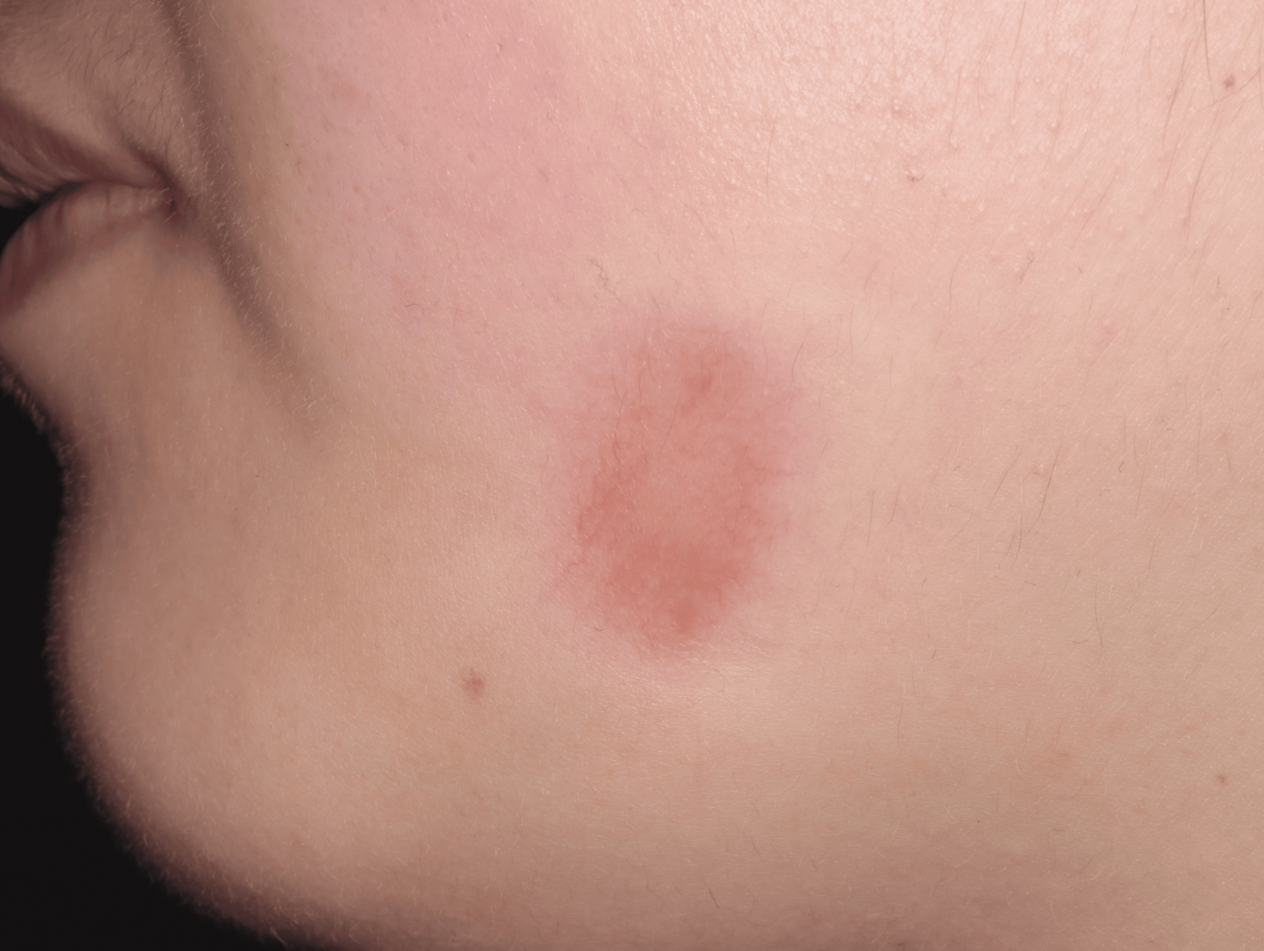
A flat and faded scar one year after the first examination.

The pregnancy progressed without complications from the dental condition, and at eight months after the first consultation, the patient delivered a healthy baby with no adverse effects from the treatments. Two months postpartum, a follow-up CT scan showed no abnormalities in the left mandibular region (Figure 6). The cutaneous lesion was a 10 mm hypertrophic scar with no other symptoms. The patient was prescribed Tranilast Capsule 100 mg (two capsules per day for 30 days) for scar management. Two months later, marking one year after the first visit, the patient remained asymptomatic, with only a faint scar remaining (Figure 7). No further treatment was needed, and routine follow-ups were scheduled to monitor for any recurrence or complications.

## Discussion

In this patient, the slight swelling at the extraction site, 8 mm periodontal pocket, and alveolar bone resorption indicate a periodontal infection in the second molar following the removal of the third molar. Wisdom tooth extraction effectively reduces biofilm accumulation surface area at the gingival interface, potentially altering subgingival anaerobic conditions, reducing pathogen colonization, and triggering immune responses to bacteria [7]. Consequently, this could lead to a chronic infection. However, current evidence lacks conclusive support that mild periodontitis alone progresses to OCF.

Given the persistent pain and swelling, we suspect the patient may have concurrent chronic periostitis, likely triggered by immunosuppression due to pregnancy. This condition, compounded by delayed healing, ultimately led to the formation of a cutaneous sinus tract (extraoral fistula). CT imaging shows a solid periosteal reaction at the left mandibular site, with an irregular cortical bone contour in the outer bone plate, suggesting chronic infection. The slight thickening of the bone plate, linked to the fistulous connection between the cutaneous lesion and the extraction site, further supports the idea that periostitis plays a role in the pathogenesis of OCF in this patient. Proliferative periostitis, also known as Garre's osteomyelitis, is characterized by new periosteal bone formation in response to inflammation, often due to adjacent odontogenic infections. As noted by Seok et al., proliferative periostitis involves localized bone formation over cortical bone, primarily stemming from dental infections, reinforcing its classification as a subtype of chronic osteomyelitis [8]. Similarly, Maurer et al. highlighted that chronic osteomyelitis underpins the understanding of proliferative periostitis, where the periosteum responds by forming new bone in reaction to underlying inflammation [9].

Furthermore, the infection occurring at the site of a lower wisdom tooth extraction progresses quietly for five months before symptoms of an external facial fistula appear. In our opinion, this is not just the common progression of OCF symptoms but also related to physiological changes during the patient's pregnancy since the recurrence of symptoms appeared more severe and related to the time of her second pregnancy. The increased estrogen levels in saliva accelerate the desquamation rate of oral mucosal cells, creating a favorable environment for bacteria to proliferate [10]. Additionally, with the increase of progesterone and estrogen during pregnancy, the maternal immune system is suppressed while the inflammatory response strengthens. Due to elevated estrogen and progesterone levels, insulin sensitivity decreases, and glucose metabolism changes during pregnancy. This leads to increased blood sugar, dilation of small capillaries, decreased elasticity of blood vessels, and increased gingival capillary permeability [11]. At the same time, the inflammatory response during pregnancy is more strongly activated, while neutrophil function is impaired, leading to increased expression of inflammatory markers [11-14]. All of the changes marked the infection susceptibility in pregnant women and may be involved in her chronic infection at the extraction site.

A pivotal understanding of the microbiological context of OCFs reveals that these infections are frequently polymicrobial [15,16]. Oral flora associated with these conditions includes both facultative anaerobes, notably varieties from the *Streptococcus* species such as *Streptococcus viridans*, and strict anaerobes such as *Fusobacterium* spp. and *Prevotella* spp. [15]. Furthermore, reports suggest various clinical contexts where *Actinomyces* spp. have been directly implicated in the formation of cutaneous fistulas. In Vandeplas et al.'s clinical case series, involving six cases of Actinomyces infections in the head and neck area after lower wisdom tooth extraction, 50% of the cases developed external facial fistulas [17]. The onset of symptoms for these cases ranged from a few days to three months after extraction, with other symptoms including induration, swelling, and pain. According to a study by Kang et al., cervicofacial actinomycosis frequently leads to the development of fistulous connections [18]. This aligns with findings by Wasyłyszyn and Borowska, who also observed that actinomycosis can result in abscesses and the associated formation of fistulae [19]. However, in our case, there was no pus or drainage at the fistula’s site, and at the extraction site, we could not conduct bacterial culture, and due to the patient’s wish, we could not perform a biopsy. Because of the lack of microbiological evidence, our treatment plan for the infection was based on the progression of symptoms and the patient’s response to broad-spectrum antibiotics.

The antibiotic treatment consists of 33 days of Amoxicillin at a dosage of 250 mg and 44 days of Clarithromycin at 200 mg, totaling 77 days of antibiotic therapy. The antibiotic treatment she received at the first dental clinic was 21 days of Amoxicillin at a dosage of 250 mg. The lesion’s size reduced at first, but after one month since stopping the use of the antibiotic, the lesion recurred with tenderness and a larger size. When she was referred to our division, Amoxicillin 250 mg was prescribed for 12 days until we had the final consultation. At the next appointment, the inflammation symptoms were relieved. However, after consultation with a dermatologist, we are still afraid of the vague reason underneath and suspect Amoxicillin resistance, which may cause the lesion to relapse like the previous treatment. We switched the antibiotic to Clarithromycin 250 mg because, based on the Guideline for Gynecological Practice in Japan, Clarithromycin can be used safely for pregnant women for longer days than Amoxicillin [20].

The effectiveness of antibiotic treatment can be limited when the underlying cause of the infection remains unaddressed and lesions persist for an extended period. Many case reports indicate that the most effective treatment for orofacial fistulas (OCF) is to eliminate the causative tooth or bacterial sources, utilizing endodontic treatment for restorable teeth and extraction for nonrestorable teeth [2,3]. In most cases, cutaneous fistulas heal uneventfully after dental treatment without the need for cosmetic surgery. Systemic antibiotics are typically unnecessary in localized infections; however, they should be considered in patients with diabetes, immunosuppression, signs of systemic infection, osteomyelitis, or *Actinomyces* spp. infection [15,16].

When actinomycosis is suspected, a long-term antibiotic regimen is essential to effectively manage the infection. The primary treatment for actinomycosis involves administering high-dose intravenous penicillin G (12-24 million U/day for adults) or ampicillin for two to six weeks. Once clinical improvement is seen, therapy should be gradually transitioned to oral penicillin V or amoxicillin, continued for 6-12 months to effectively prevent relapse [17,21]. In the case of penicillin hypersensitivity, other drugs should be considered such as clindamycin, macrolides (erythromycin, clarithromycin, or azithromycin), doxycycline, tigecycline, and chloramphenicol [21]. 

Given the concurrent proliferative periostitis (a subtype of chronic osteomyelitis) in this case, the management strategy involved the use of appropriate antibiotic therapy for this patient's condition. In osteomyelitis cases, the antibiotics need an excellent penetration into the bone and joint tissue to eradicate the microorganism [22]. A literature review studied more than 30 antibiotics, and the results show that almost all antibiotics have good penetration in bone and joint tissues [23]. Studies on prolonged antibiotic treatment suggest that antibiotics can be used to control infections in patients who are not suitable for more invasive surgical methods, which is especially relevant in this pregnant patient [24,25]. Since 1970, Waldvogel et al. have recommended at least four weeks of therapy [26], while randomized controlled trials and retrospective studies have proposed durations ranging from four to six weeks [22,27]. Until now, the optimal duration of treatment is still not universally agreed upon [22], and deciding on the optimal treatment duration requires balancing the goal of effective bacterial eradication with minimizing potential side effects, the risk of antibiotic resistance, and the financial implications of extended treatment [20]. In this context, the progression of the lesion is a key factor in determining treatment duration. When a stable and positive reduction in the lesion's size is observed, and there are no further symptoms, antibiotic treatment can be discontinued. However, we believe that the treatment outcome was more strongly influenced by periodontal scaling and the physiological changes in inflammatory and immune responses during pregnancy. The use of long-term antibiotics in this pregnant patient was not aimed at completely eradicating the infection but at symptom management and preventing progression or recurrence to a clinically significant level [16,17]. The use of long-term antibiotics in this pregnant patient was not intended to cure the infection completely, but to manage symptoms and prevent progression or recurrence to a clinically significant level [24,25].

This case report, while valuable in demonstrating the management of OCF in a pregnant patient, has some limitations. First, the study is based on a single case, limiting the generalizability of the findings to a broader population. The outcomes observed may not apply to all patients with similar conditions. Additionally, the lack of a control group means that the effectiveness of the treatment regimen, particularly the prolonged antibiotic therapy, cannot be definitively compared to other possible treatment options. Another limitation is the absence of long-term follow-up data beyond the one-year mark, which restricts understanding of the potential for recurrence or long-term effects of the treatment. Furthermore, while the pregnancy was managed without complications, the impact of the antibiotic regimen on the fetus remains unquantified in this case, requiring caution when generalizing these findings to other pregnant patients.

To improve the management of OCF, especially in pregnant patients, clinicians should maintain a high index of suspicion for OCF in patients presenting with cutaneous lesions near the oral cavity, especially if there is a history of dental issues. Early diagnosis can prevent unnecessary treatments and prolonged antibiotic use. A multidisciplinary approach involving dermatologists, dentists, maxillofacial surgeons, and obstetricians addresses dental and pregnancy-related changes. Treatment plans should be tailored to the patient's pregnancy status, favoring non-invasive methods like periodontal scaling to manage symptoms while minimizing risks. Educating patients on oral hygiene and the signs of OCF is essential, along with regular follow-up care to monitor infection resolution and manage any complications. Postpartum follow-up is particularly important to ensure complete healing and address any residual issues. By implementing these recommendations, we believe that healthcare providers can improve the management of OCF, reduce the risk of misdiagnosis, and enhance patient outcomes. In this case, we considered that post-extraction infection occurred during the healing process and that a decline in systemic condition due to pregnancy-related nausea prolonged the infection, ultimately leading to a cutaneous fistula. Long-term antibiotic therapy and hormonal changes following childbirth were thought to have contributed to the improvement of the cutaneous fistula. Furthermore, when planning for pregnancy and considering wisdom tooth extraction, we advise scheduling the procedure before conception at six months. This approach allows ample time for healing and reduces potential complications during early pregnancy [20].

## Conclusions

OCF is a rare but significant condition that is often misdiagnosed due to its diverse clinical presentations and insidious onset. This case highlights the complexity of diagnosing and treating OCF, especially in a pregnant patient where physiological changes can exacerbate the condition. In this case, we considered that post-extraction infection occurred during the healing process and that a decline in systemic condition due to pregnancy prolonged the infection, ultimately leading to a cutaneous fistula. Antibiotic therapy and hormonal changes following childbirth were thought to have contributed to the improvement of the cutaneous fistula. Prolonged antibiotic therapy, aimed at managing symptoms and preventing progression rather than curing infection, emphasizes the importance of identifying and addressing underlying dental causes.
